# Differential Regulation of Transcription Factors by Location-Specific EGF Receptor Signaling via a Spatio-Temporal Interplay of ERK Activation

**DOI:** 10.1371/journal.pone.0041354

**Published:** 2012-09-12

**Authors:** Peng Wu, Ping Wee, Jennifer Jiang, Xinmei Chen, Zhixiang Wang

**Affiliations:** The Department of Medical Genetics and Signal Transduction Research Group, Faculty of Medicine and Dentistry, University of Alberta, Edmonton, Alberta, Canada; University of Edinburgh, United Kingdom

## Abstract

It is well established that EGFR signals from both the plasma membrane (PM) and endosome (EN). However, very little is known about whether and how the EGFR signals at the PM and EN to differentially regulate various signaling pathways and the physiological outcomes. In this communication, we established a system that allowed the specific activations of EGFR at different cell locations: PM and EN. PM activation of EGFR is achieved by activation of endocytosis-deficient mutant EGFR1010LL/AA stably expressed in CHO cells (CHO-LL/AA cell). EN activation of EGFR is achieved by activating the wild type EGFR stably expressed in CHO cells (CHO-EGFR cell) after its internalization into EN with a previously reported protocol. We showed that both EGFR activations at PM and EN activated ERK to a similar level, but differentially stimulated transcriptional factors c-jun and c-fos. We further showed that EGFR activations at PM and EN resulted in differential spatio-temporal dynamics of phosphorylated ERK which caused the differential activation of two downstream substrates ELK1 and RSK. Finally we showed that EGFR signaling from PM and EN led to different physiological outcomes. CHO-LL/AA cells that only generate PM EGFR signals have a larger cell size and slower proliferation rate than CHO-EGFR cells. We conclude that location-specific EGFR activation differentially regulates cell functions through a spatio-temporal interplay of ERK activation.

## Introduction

Activation of epidermal growth factor (EGF) receptor (EGFR) by EGF stimulates various signal transduction pathways leading to cell mitogenesis and survival [1]–[3]. Perturbation of EGFR signaling by mutations and other genetic alterations contributes to the development of human cancers [2], [4], [5]. The binding of EGF to EGFR at the plasma membrane (PM) induces the dimerization of EGFR, which results in the activation of the EGFR tyrosine kinase and its trans-autophosphorylation. The sites of tyrosine phosphorylation in the activated EGFR form signaling complexes with many signaling proteins including Grb2, SHC, phospholipase C-γ1 (PLC-γ1), the p85α subunit of PI3K (p85), p120 Ras GAP, Src, and Cbl [6]–[8]. The formation of the receptor-signaling protein complexes then initiates the activation of various signaling pathways. For example, the interaction between EGFR and SHC/Grb2 results in the recruitment of Sos to the plasma membrane to activate Ras. Ras then activates Raf , which leads to the activation of MEK and ERK [3].

The ligand-bound receptors are also rapidly internalized into endosomes (EN) and eventually degraded in lysosomes [3], [9]. It is well-established that endocytosis of the EGFR from PM to EN and then lysosomes results in the degradation of the receptor, which can attenuate receptor signaling and may be conceived of as a tumor suppressor pathway [10]–[14]. However, accumulated evidence also suggests that internalized EGFR is phosphorylated, catalytically active, binding to various signaling molecules, activating various signaling pathways and leading to cell proliferation and survival [11], [15]–[23]. More importantly, EGFR signaling from EN may regulate cell signaling differently from EGFR signaling from PM [24]–[32]. Thus, subcellular localization of activated EGFR through endocytosis provides another layer of regulation of EGFR-mediated cell signaling. Recently, EGFR signaling from other subcellular locations such as the nucleus and mitochondria has been reported [33], [34]. Moreover, the spatial dynamics of many signaling molecules, including Ras, Rap1, ERK and phosphatidylinositol-3,4,5-triphosphate, have been shown to play significant roles in regulating various functions in the cell [35]–[38].

We have shown that specific EGFR signaling from EN, similar to standard EGFR signaling that includes both PM and EN EGFR signaling, is sufficient to activate major signaling pathways and lead to cell proliferation and survival [22], [23], [39]. On the other hand, extensive studies have been conducted to determine whether distinct subcellular localization of activated EGFR will generate different cellular signaling. Many studies showed that the various signaling proteins, especially the signaling proteins in the ERK pathway, are differentially activated by EGFR signaling generated from PM and EN [29]–[32]. However, the results are very controversial and sometimes contradictory as to the net effects on ERK signaling.

The lack of definite results is partially due to the lack of a proper system to generate specific EGFR signaling from EN and PM. Many studies have attempted to define the differences of EGFR signaling between PM and EN. However, some data were only based on the inhibition of EGFR endocytosis [29]–[31], [40], [41], and thus the results only reflect the differences between PM EGFR signaling and standard EGFR signaling (including both PM and EN EGFR signaling). The other data were based on isolation of endosomes following EGFR stimulation [24]–[28]. In these cases EGFR was activated from the PM and thus the activation of downstream signaling proteins may be due to both PM and EN EGFR signaling. No direct comparison has ever been made between specific PM signaling and EN signaling due to the lack of suitable cell systems. We have established a system that generates specific EGFR signaling from EN [22], [23], [39]. Briefly, we treated cells expressing wild type EGFR with EGF in the presence of AG1478, an EGFR tyrosine kinase inhibitor, and monensin, an inhibitor of EGFR recycling. This treatment led to the internalization of inactive EGF-EGFR complexes into endosomes. The endosome-associated EGFR was then activated by removing AG1478 with washing [22], [39]. Recently, we generated an endocytosis-deficient mutant EGFR1010LL/AA with the mutation of the di-leucine motif ^1010^LL^1011^ to Alanines [42]. We showed that the di-leucine ^1010^LL^1011^ is required for EGF- induced rapid internalization of full length EGFR and that the role of ^1010^LL^1011^ in EGFR internalization is independent of EGFR kinase activation [42]. As this mutation is outside of the kinase domain and not at the tyrosine phosphorylation sites, this mutant may be fully activated by EGF and persistently generate PM EGFR signaling. We have indeed showed that this mutant EGFR1010LL/AA is strongly phosphorylated by EGF [42]. Thus we are in a position to directly compare EN EGFR signaling with PM EGFR signaling to understand how location-specific EGFR activation regulates cell signaling.

In this communication, using our previously established CHO cell lines stably expressing YFP-tagged wild type EGFR (CHO-EGFR cell) or mutant EGFR1010LL/AA (CHO-LL/AA cell) [42], we showed that in response to EGF, EGFR1010LL/AA is not internalized but is phosphorylated at all of the major tyrosine sites with a strength similar to the standard activation of wild type EGFR. This suggests that we have a system to generate competent EGFR signaling specifically from the cell surface. This system together with our previously established system that specifically activates EGFR in EN allowed us to directly compare PM EGFR signaling with EN EGFR signaling. We showed that location-specific EGFR activation at both PM and EN stimulated ERK activation to a similar level, but differentially regulated transcriptional factors c-jun and c-fos. We further showed that EGFR activations at PM and EN resulted in differential spatio-temporal dynamics of phosphorylated ERK. PM EGFR activation resulted in a gradual and lasting build up of pERK in nuclear fractions, but EN EGFR activation resulted in a quick and transient build-up of pERK in nuclear fractions. The differential spatio-temporal dynamics of phosphorylated ERK cause the differential activations of two downstream substrates ELK1 and RSK. Finally we showed that EGFR signaling from PM and EN leads to different physiological outcomes. CHO-LL/AA cells that only generate PM EGFR signals have a larger cell size and slower proliferation rate than CHO-EGFR cells.

## Materials and Methods

### Antibodies and Chemicals

All the primary antibodies were from Santa Cruz Biotech (Santa Cruz, CA). The horseradish peroxidase(HRP)- conjugated secondary antibodies were from Bio-Rad (Hercules, CA) and the rhodamin-conjugated secondary antibodies were from Jackson ImmunoResearch (West Grove, PA). The oligos used for reverse transcription (RT-PCR) and the cell culture reagents were from Invitrogen (Carisbad, CA). Unless otherwise specified, all other chemicals were from Sigma (St. Louis, MO).

### Cell culture

The cell lines used in this research included 293T cells, CHO cells (Chinese hamster ovary cell, gift from Dr. Luc Berthiaume, University of Alberta), our previously selected CHO cells stably expressing YFP-tagged wild-type EGFR (CHO-EGFR cells), and our endocytosis-deficient mutant EGFR1010LL/AA (CHO-LL/AA cells) [42]. All of the above cells were grown at 37°C in Dulbecco's modified Eagle's medium (DMEM) containing 10% FBS, and penicillin-streptomycin (100 U/ml) and were maintained in a 5% CO_2_ atmosphere. G418 was added to a final concentration of 500 µg/ml to maintain CHO-EGFR and CHO-LL/AA cells.

### Cell treatment with EGF to generate location-specific EGFR activation

Two different treatment methods are used. For standard treatment, cells were serum starved for 24 h, and then stimulated with EGF with a final concentration of 50 ng/ml for the indicated time. In CHO-LL/AA cells, standard treatment of EGF initiates EGFR activation at the plasma membrane only (PM activation of EGFR). In CHO-EGFR cells, standard EGF treatment results in both EGFR activation at plasma membrane and in endosomes, which we used as the control (SD activation of EGFR). The specific activation of EGFR in endosome (EN activation of EGFR) was achieved by our previously described method [22]. Briefly, CHO cells expressing wild type EGFR were serum starved for 24 h, and were pretreated with 0.5 µM AG1478 for 15 min. Monensin and EGF were then added to a final concentration of 100 µM and 50 ng/ml, respectively. After incubating at 37°C for 30 min, cells were washed with PBS for at least 5 times to remove AG1478 and monensin, which is designated as time 0. Washed cells were then incubated in 37°C for the indicated time.

### Subcellular fractionation

Two types of subcellular fractionation were performed. In some experiments, cell homogenates were separated into PM, EN and cytosolic fractions. In other experiments, cell homogenates were separated into nuclear and non-nuclear fractions. Isolation of plasma membrane (PM), endosomal (EN), and cytosolic (CY) fractions was carried out by our previously described method [22], [43]. Briefly, following treatment cells were scraped into homogenization buffer (0.25 M sucrose, 20 mM Tris-HCl, pH 7, 1 mM MgCl_2_, 4 mM NaF, 0.5 mM Na_3_VO_4_, 0.02% NaN_3_, 0.1 mM 4-(2-aminoethyl)-benzenesulfonyl fluoride, 10 µg/ml aprotinin, 1 µM pepstatin A) and homogenized. The homogenates were centrifuged at 200× g for 5 min to remove cell debris and nuclei (P1). The post nuclear supernatant (S1) was then centrifuged at 1500× g for 10 min to yield a supernatant (S2) and a pellet (P2). Next, P2 was re-suspended in homogenization buffer (0.25 M sucrose), overlaid upon an equal volume of 1.42 M sucrose buffer and centrifuged at 82, 000× g for 1 h. The pellicule at the 0.25–1.42 M interface was collected as the PM fraction. The S2 fraction was centrifuged at 100 000× g for 30 min to yield the soluble CY fraction and a microsomal pellet. This pellet was re-suspended in 0.25 M sucrose buffer and overlaid upon a discontinuous sucrose gradient containing equal volumes of homogenization buffer at 1.00 and 1.15 M sucrose. The re-suspension was centrifuged at 200 000× g for 1.5 h to obtain the purified EN fraction at the 0.25–1.00 M interface. For a typical experiment, the total yielding is 30 µg for the plasma membrane, 30 µg for the endosome fraction and 1 mg for the cytosol fraction. The total yielding of each fraction was very consistent for all of the treatment.

To isolate nuclear and non-nuclear fractions, after treatment, cells were first suspended in homogenization buffer and homogenized for 30 times with a homogenizer. The nuclei were then separated from homogenate by spinning down at 200× g for 10 min twice. The supernatant was then centrifuged at 14 000× g for 10 min to spin down contaminating nuclei and cell debris. The supernatant was then saved, which contained cytoplasm and cell membrane. The pellet of the first centrifugation was suspended in homogenization buffer and was then centrifuged at 200× g for 10 min for at least 3 times to remove cytoplasmic contaminations. The pellets were then suspended in M-Per and used as nuclear extracts.

### Immunoblotting

Immunoblotting was performed as previously described [22]. Briefly, protein samples were separated by SDS-PAGE and then were transferred onto nitrocellulose and probed with primary antibodies. The primary antibodies were detected with a HRP-conjugated secondary antibody followed by enhanced chemiluminescence development (Pierce Chemical, Rockford, IL) and light detection on Fuji Super RX Film (Tokyo, Japan). For graphical analysis, sub-saturated band exposures were scanned using a GS-800 calibrated densitometer. Quantification of band intensity was finished by using ImageJ software. For the quantification of the phosphorylation of various proteins, the band intensity of phosphorylated proteins is normalized against the band intensity of non-phosphorylated proteins. Two-tailed *student t-tests* were completed using MedCalc software.

### Fluorescence Microscopy

Cells were grown on glass coverslips to about 80% confluence, and were then serum starved for 24 h. After treatment, the cells were fixed by −20°C methanol or 4% paraformaldehyde for 15 min at room temperature and permeabilized with 0.2% PBS-Triton X-100 for 20 min. Then, the cells were incubated with the indicated primary antibodies at room temperature for 1 h or 4°C overnight, followed by incubation with FITC/TRITC-conjugated secondary antibodies for 1 h at room temperature. 300 nM DAPI was used to stain the nuclei of cells for 2 minutes. The stained cells were then observed and photographed with an inverted Olympus IX71 microscope (Applied Precision) with standard filters, and the data were analyzed using Delta Vision softWoRx software.

### RT-PCR

RNA purification was performed using TRIzol® reagent from Invitrogen Co. In brief, cells were dissolved in 1 ml TRIzol® reagent, and then incubated at room temperature for 5 min. 0.2 ml of chloroform was added to the homogenized sample and the sample was incubated at room temperature for 2 to 3 minutes before being centrifuged at 12,000× g for 15 minutes at 4°C. The aqueous phase was transferred to a fresh tube and 0.5 ml of isopropyl alcohol was added. The sample was then incubated at room temperature for 10 min before centrifugation at 12,000× g for 10 minutes. Next, the supernatant was discarded and the RNA pellet was washed once with 75% ethanol. Then, the RNA was dissolved in RNase-free water. The purified RNA was then used for RT-PCR. Briefly, the first-strand cDNA was synthesized in the presence of 5 ml 10× MMLV RT Buffer, 5 ml 0.1 M DTT, 10 ml dNTP Mix (2.5 mM each dNTP), 0.5 mg oligo(dT), 1 mg poly-A^+^ selected mRNA, and 10 U MMLV RT at 37°C for 60 minutes. The synthesized DNA was then denatured at 95°C and placed on ice. The DNA was then amplified by PCR with 25 cycles of denaturation for 30 seconds at 95°C; annealing for 45 seconds at 60°C; and extension for 60 seconds at 72°C.

### Cell Proliferation Assay and cell size quantification

Two methods were used to determine the cell proliferation rate, BrdU incorporation and cell counting. For cell counting, CHO-EGFR, CHO-LL/AA and transfected 293T cells (approximately 1×10^5^ cells/well) were plated in six-well tissue culture plates and cultured in a CO_2_ incubator. The number of cells in the designated areas of the plates was recorded every 24 hours for 2 days. Cell proliferation is measured by the fold of increase in cell numbers. BrdU incorporation was performed using BrdU cell proliferation assay kit (Exalpha Biologicals, Inc. USA) according to manufactuer's instruction. The cell size was determined by a well established confocal microscopic method [44]. We measured diameter of each cell at three planes, apical, middle and basal. For the diameter of each plane, we measured both the longest and shortest cross lines of the cell, then used the average of these two lines as the diameter. The diameter of the cell is the average of the diameters of three planes. For each value, 30 cells were measured.

## Results

### Location-specific EGFR activation

After establishing a system to specifically activate EGFR in EN [22], [39], we focused our studies on establishing a system to specifically activate EGFR at the PM without a broad impact on general cell function. We recently identified an endocytosis-deficient mutant EGFR1010LL/AA with mutation of the dileucine motif ^1010^LL^1011^ to alanines and showed that this mutant is strongly phosphorylated by EGF [42]. Thus it is likely that this mutant will allow us to establish a system to specifically generate persistent EGFR signaling only at PM.

**Figure 1:**
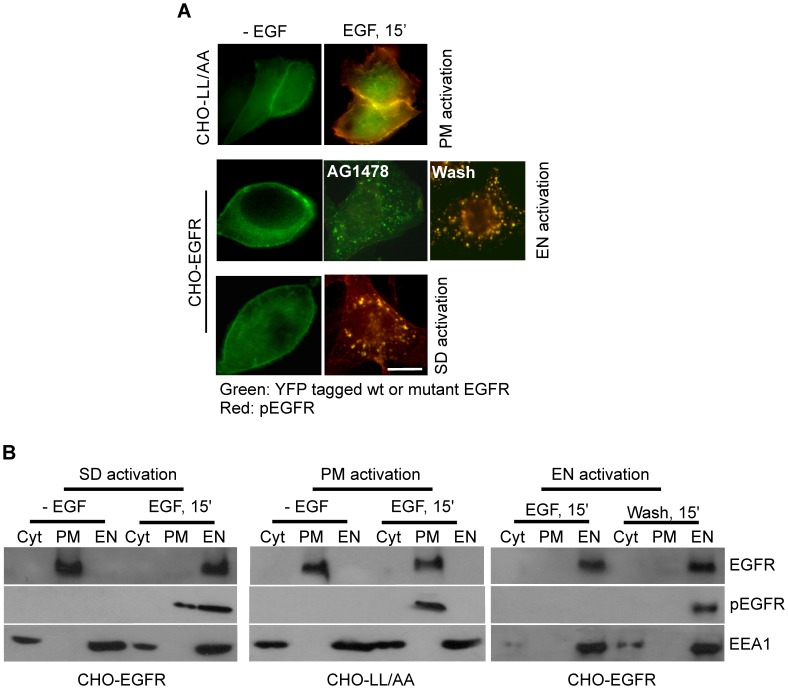
Location-specific activation of EGFR. CHO-EGFR and CHO-LL cells were treated to specifically activate EGFR at PM or EN as described in Experimental procedures. SD activation of EGFR is used as a control. (A) Location-specific activation of EGFR was examined by fluorescence microscopy. The localization of EGFR (green) is revealed by the intrinsic fluorescence of tagged YFP and the localization of pEGFR (red) is revealed by indirect immunofluorescence as described in Experimental procedures. Size bar, 10 µm. (B) Location-specific activation of EGFR was examined by subcellularly fractionation. Following the location-specific EGFR activation, cells were subcellular fractionated into the plasma membrane (PM), endosome (EN) and cytosol (CY) fractions. The subcellular fractions were subjected to immunoblotting with anti-EGFR, anti-pTyr and anti-EEA1 antibodies.

Indeed, we showed that EGF stimulation of CHO-LL/AA cells resulted in plasma membrane-specific EGFR activation (referred as PM activation) since mutant EGFR1010LL/AA was not internalized in response to EGF (Fig. 1A). The specific activation of EGFR in endosomes (referred as EN activation) was achieved in CHO-EGFR cells expressing wild type EGFR by previously established methods [22], [39]. Briefly, we treated CHO-EGFR cells with EGF in the presence of AG1478, an EGFR tyrosine kinase inhibitor. This treatment led to the internalization of inactive EGF-EGFR (wild type) complexes into endosomes. The endosome-associated EGFR was then activated by removing AG1478 with washing (Fig. 1A). Standard EGFR activation (referred as SD activation), serving as a control, was achieved by normal EGF stimulation of wild type EGFR in CHO-EGFR cells. SD EGFR activation includes both the initial PM activation and the later EN activation of EGFR (Fig. 1A).

The location-specific activation of EGFR in various CHO cell lines was verified by subcellular fractionation and immunoblotting. As shown in Fig. 1B, activated EGFR was exclusively localized at the PM fraction, not in endosomes following PM activation of EGFR in CHO-LL/AA cells; activated EGFR was exclusively localized in endosomes, not at the PM fraction following EN activation of wild type EGFR in CHO-EGFR cells. EEA1 was used as a marker for endosomes. These results indicate that we have successfully established a system to specifically activate EGFR in specific locations: PM and EN.

### Characterization of location-specific activated EGFR

**Figure 2:**
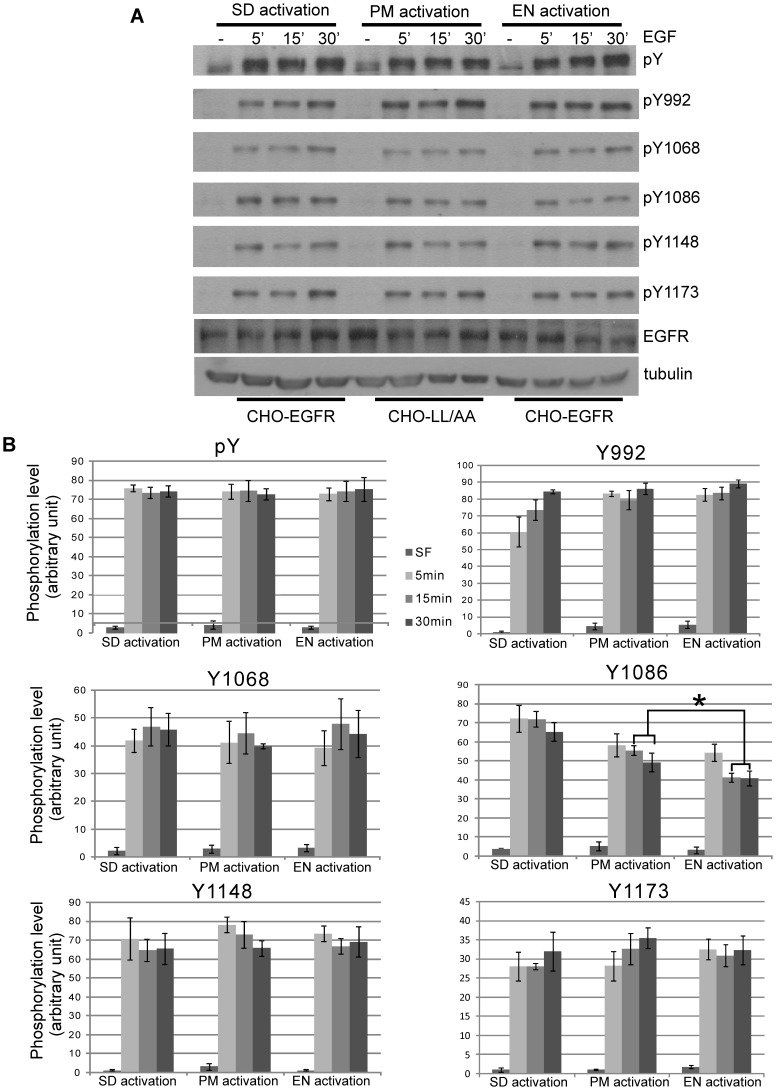
Characterization of EGFR phosphorylation following location-specific activation. CHO-EGFR and CHO-LL cells were treated to specifically activate EGFR at PM or EN for the indicated time as described in Experimental procedures. (A) The phosphorylation of EGFR was examined with antibody to phosphotyrosine (pY99) and various antibodies specific for the 5 major tyrosine phosphorylation sites as indicated. (B) Quantification of the data from (A). Each value is the average of at least three independent experiments and the error bar is the standard error. * indicates that the difference is statistically different (p<0.05).

We examined whether EGFR is similarly activated under PM, EN and SD activation. The activation of EGFR was assessed by the overall phosphorylation of EGFR using a general antibody to phosphorylated tyrosine (pY) and by the specific phosphorylation of the five known major tyrosine residues: Y992, Y1068, Y1086, Y1148 and Y1173 using specific antibodies. The overall phosphorylation and the phosphorylation of each of these five tyrosine residues except for Y1086 were very similar following PM, EN and SD activation (Fig. 2A&B). For Y1086, EN activation of EGFR induced weak phosphorylation when compared with SD and PM activation of EGFR (Fig. 2A–B). Statistical analysis indicated that this difference is significant (p<0.05).

**Figure 3:**
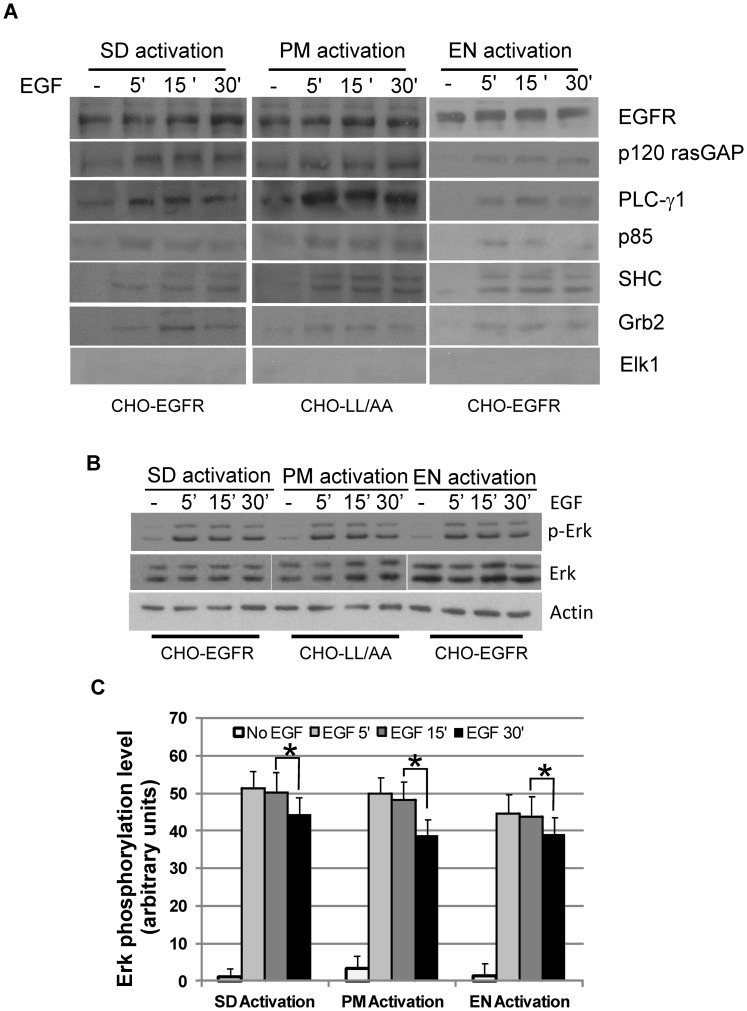
The association of EGFR with signaling proteins and the activation of ERK. (A) Co-immunoprecipitation (co-IP) of EGFR with downstream signaling proteins following location-specific EGFR activation in various CHO cells. Following location-specific EGFR activation, wild type EGFR or mutant EGFR1100LL/AA was immunoprecipitated from cell lysates and the co-IP of downstream proteins was examined by immunoblotting with various antibodies as indicated. (B) Activation of ERK following location-specific activation of EGFR in CHO cells. ERK activation was examined by immunoblotting with antibody to phosphorylated ERK (pERK). (C) Quantification of the data from (B). Each value is the average of at least three independent experiments and the error bar is the standard error. * indicates that the difference is statistically different (p<0.05).

We further examined whether the activation of EGFR at various locations stimulates the association of EGFR with various signaling proteins including Grb2, SHC, PLC-γ1, p120 rasGAP, p85 and Cbl by co-immunoprecipitation (co-IP). EGFR1010LL/AA from CHO-LL/AA cells and wild type EGFR from CHO-EGFR cells were IPed with an antibody to EGFR following PM, EN and SD activation of EGFR. As a control, we IPed EGFR with normal rabbit serum and no detectable EGFR was IPed (data not shown). The co-IP of the various signaling proteins was examined by immunoblotting of the immunoprecipitates with various antibodies. We showed that signaling proteins including Grb2, SHC, p120 rasGAP, p85 and Cbl bound to EGFR1010LL/AA following PM activation similarly to wild type EGFR following both EN and SD activation (Fig. 3A). However, PLC-γ1 bound to EGFR1010LL/AA following PM activation more strongly than wild type EGFR following EN and SD activation (Fig. 3A). As a control we blotted the immunoprecipitates with an antibody to ELK1 and no co-IP of ELK1 was detected. Together, these data indicate that in general, the activation of EGFR at both EN and PM results in its phosphorylation in all of the major tyrosine residues and its association with most downstream signaling proteins.

### Phosphorylation of ERK by location-specific EGFR activation

We next examined the effects of the location-specific EGFR activation on the activation of ERK1/2 as the data published so far has been very controversial. As shown in Fig. 3B&C, under all three conditions, ERK1/2 was similarly activated. ERK1/2 was quickly phosphorylated following the activation of EGFR at various locations. ERK1/2 phosphorylation intensity reached maximum at 5 to 15 min, and was still high at 30 min, but lower than that at 5–15 min (Fig. 3B). This decrease is statistically significant (p<0.05) (Fig. 3C). These data suggest that EGFR signaling generated from PM and EN are similar in terms of ERK phosphorylation level and time course.

### Differential phosphorylation and expression of transcription factors by location-specific EGFR signaling

**Figure 4:**
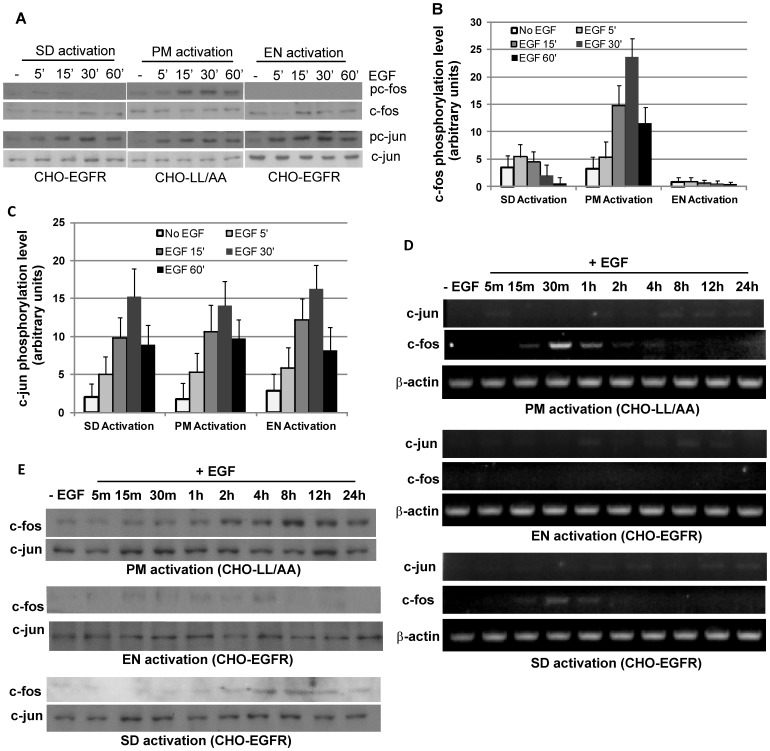
Activation and expression of transcription factors c-jun and c-fos. (A) Activation of c-jun and c-fos following location-specific activation of EGFR. The activation of c-jun and c-fos was examined by immunoblotting with antibodies to phosphorylated c-jun (pc-jun) and c-fos (pc-fos). (B) Quantification of c-fos activation with the data from (A). Each value is the average of at least three independent experiments and the error bar is the standard error. (C) Quantification of c-jun activation with the data from (A). Each value is the average of at least three independent experiments and the error bar is the standard error. (D) mRNA transcription of c-fos and c-jun following location-specific EGFR activation. mRNA transcription of c-fos and c-jun was determined by RT-PCR as described in Experimental procedures. (E) Expression of c-fos and c-jun following location-specific EGFR activation. The expression of c-fos and c-jun was determined by immunoblotting with antibodies to c-fos and c-jun.

We next looked at the signaling events further down the cascades. We examined whether the location-specific EGFR signaling differentially regulates the transcription factors c-fos and c-jun in terms of their phosphorylation and expression. We first examined the phosphorylation of c-fos and c-jun by immunoblotting. We showed that c-fos was phosphorylated by PM EGFR activation in CHO-LL/AA cells following EGF stimulation, but no phosphorylation of c-fos was detected in CHO-EGFR cells following EN activation of EGFR (Fig. 4A&B). As a control, SD activation of EGFR caused c-fos activation, but much weaker than that following PM activation of EGFR (Fig. 4A&B). These data indicate that PM EGFR activation, but not EN EGFR activation, specifically induces the phosphorylation of transcriptional factor c-fos.

We then determined the phosphorylation of c-jun in response to location-specific EGFR activation (Fig. 4A&C). We showed that under all three conditions, c-jun was phosphorylated following EGF stimulation for 5 min and the phosphorylation level continued to increase and remained high afterwards. No significant differences in c-jun phosphorylation were observed following the activation of EGFR at various locations.

We further examined whether the location-specific EGFR activation differentially regulates the expression of c-fos and c-jun. We first examined the mRNA level of c-fos and c-jun by RT-PCR. As shown in Fig. 4D, In CHO-LL/AA cells, PM activation of EGFR for 15 min significantly increased the mRNA level of c-fos. The c-fos mRNA level reached maximum at 30 min EGF addition and then gradually decreased in next hour to a lower but still significant level that was maintained for more than 4 hours. This indicates that PM EGFR signaling strongly stimulated c-fos transcription. On the other hand, we did not observe detectable c-fos mRNA following the EN activation of EGFR. SD activation of EGFR resulted in the transcription of c-fos mRNA, but at a much lower level than that following PM activation of EGFR. These data showed that activation of EGFR at different subcellular location has different effects on c-fos mRNA transcription. We only observed very weak increases of c-jun mRNA following PM, EN and SD activation of EGFR (Fig. 4D). This weak increase of c-jun mRNA was very similar following PM, EN and SD activation of EGFR.

We also examined the expression level of transcription factors at the protein level (Fig. 4E). Similar to our observation of mRNA, the expression of c-fos was stimulated by PM activation of EGFR, but not EN activation of EGFR. SD activation of EGFR slightly increased c-fos expression. Interestingly, the basal level of c-fos expression in the absence of EGF stimulation is also significantly higher in CHO-LL/AA cells than in CHO-EGFR cells, which suggests that chronic basal EGFR activation from PM may enhance c-fos expression in the absence of acute PM activation of EGFR. On the other hand, c-jun expression levels were not changed in response to PM, EN and SD activation of EGFR.

### Nuclear translocation of phospho-ERK following location-specific EGFR activation

Our above results showed that both PM and EN activation of EGFR resulted in similar ERK1/2 phosphorylation level, however, location-specific activation of EGFR differentially regulated the activation and expression of transcription factors especially c-fos. We next tried to determine the molecular mechanism that connect these two seemingly contradictory outcomes. It has been shown that in addition to elevated ERK activity, nuclear translocation of ERK is also required for cell proliferation [45]. We therefore examined whether location-specific EGFR activation leads to differential nuclear translocation of pERK.

**Figure 5:**
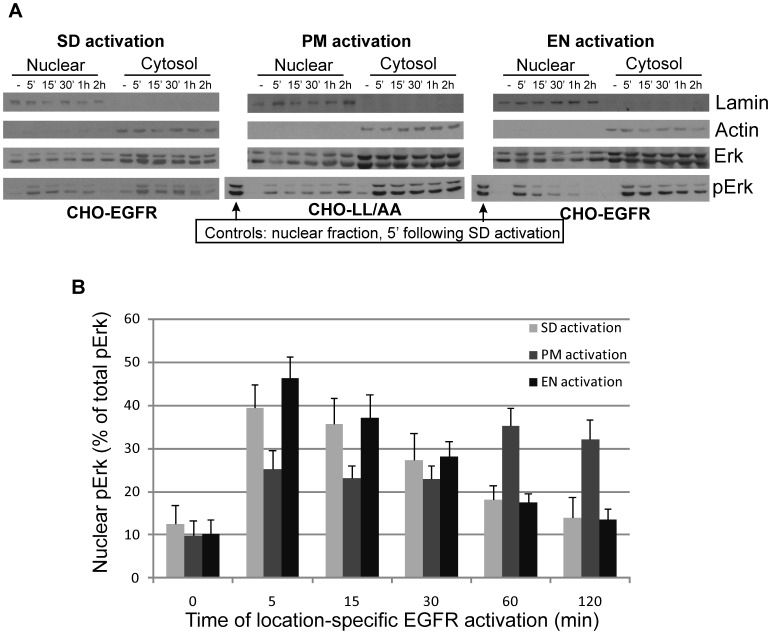
Spatio-temporal activation of ERK. (A) Following location-specific activation of EGFR for indicated time, the spatio-temporal activation of ERK was determined by subcellular fractionation followed by immunoblotting with anti-pERK and anti-ERK antibodies as described in Experimental procedures. Lamin was used as a marker for the nuclear fraction and actin was used as a marker for the non-nuclear fraction. (B) Quantification of ERK activation with the data from (A). The ERK activation level is normalized against the level of total ERK. Each value is the average of at least three independent experiments and the error bar is the standard error.

Nuclear translocation of pERK was first determined by subcellular fractionation. Following the location-specific EGFR activation in CHO-EGFR and CHO-LL/AA cells for indicated time, the cell homogenates were subcellularly fractionated into nuclear and non-nuclear fractions. Actin was used as a marker for non-nuclear fractions and Lamin was used as a marker for nuclear fractions. In Fig. 5, we showed by immunoblotting that PM activation of EGFR resulted in a gradual and lasting build up of pERK in nuclear fractions, but EN activation of EGFR resulted in a quick and transient build-up of pERK in nuclear fractions. At 5 min, more pERK was translocated to nuclear fractions following EN activation than that following PM activation. However, at 60 min, nuclear pERK is significantly higher following PM activation than that following EN activation. These data indicated that location-specific EGFR activation resulted in the distinct spatio-temporal distribution of pERK. PM activation of EGFR resulted in a slow but steady build-up of pERK in the nucleus, however, EN activation of EGFR caused a quick, but brief nuclear localization of pERK.

**Figure 6:**
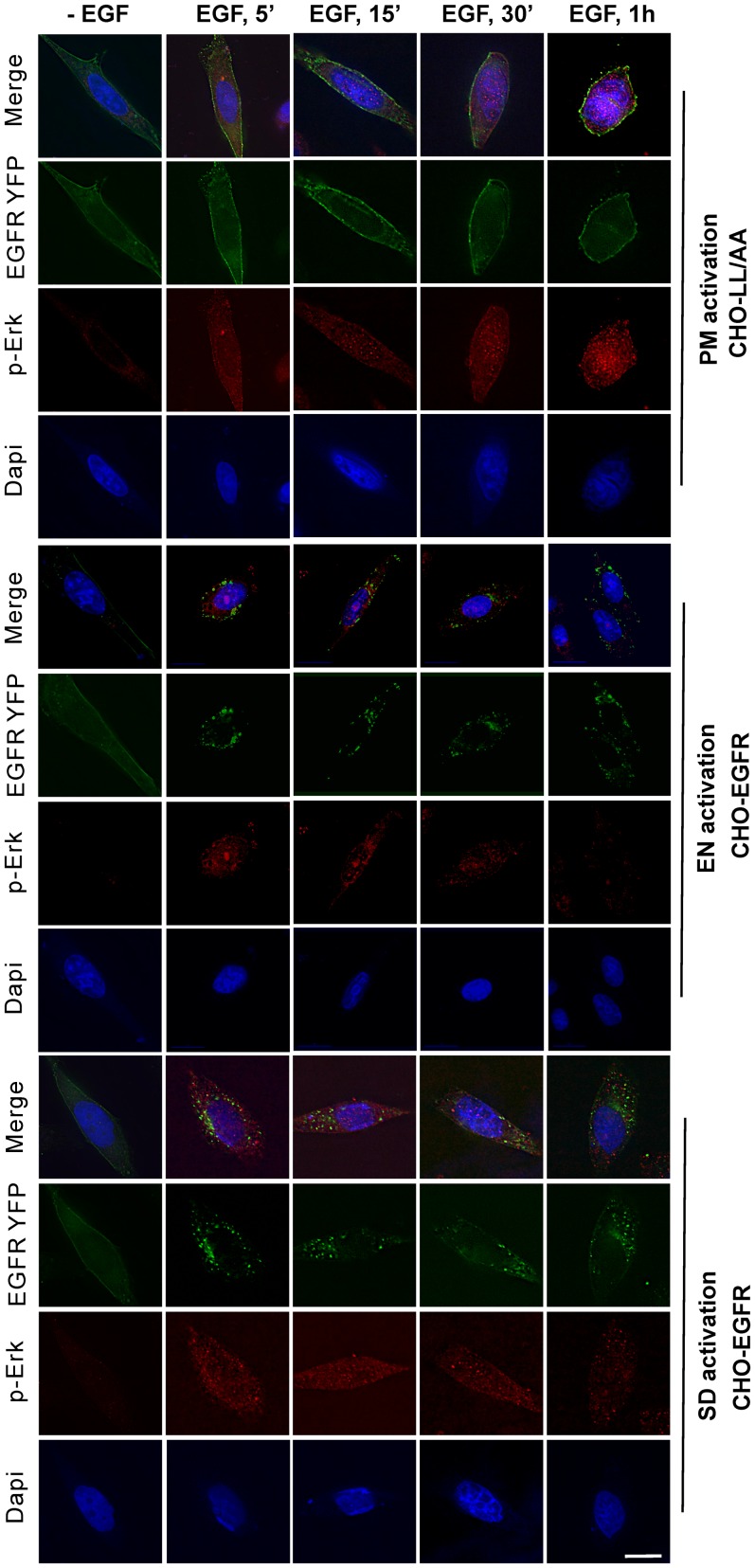
Activation and translocation of ERK. Following PM, EN and SD activation of EGFR for the indicated times in CHO stable cell lines, the activation and translocation of ERK (red) were examined by indirect immunofluorescence with an antibody to pERK followed by TRITC conjugated secondary antibody. EGFR localization (green) was revealed by the intrinsic fluorescence of tagged YFP and the nucleus was stained by Dapi (blue). Size bar, 10 µm.

We further studied the spatio-temporal distribution of pERK by indirect immunofluorescence following location-specific EGFR activation in both CHO-EGFR and CHO-LL/AA cells (Fig. 6). The localization of EGFR was viewed by the intrinsic fluorescence of tagged YFP (green), the localization of pERK was revealed by primary anti-pERK antibody followed with TRITC conjugated secondary antibody (red), and the nucleus was marked by Dapi stain (blue). As shown in Fig. 6, in CHO-LL/AA cells, following PM activation of EGFR for 5 min, pERK was mostly localized to the peripheral region of the cell. Some pERK was co-localized with EGFR at PM, but no significant nuclear localization of pERK was detected. At 15 min, significant amount of pERK was still localized to PM and the peripheral region of the cell, but nuclear localization of pERK was increased. At late time points, nuclear localization of pERK was steadily built up and at 1 h pERK was mostly localized to the nucleus. However, EN activation of EGFR in CHO-EGFR cells resulted in a quick, but brief nuclear localization of pERK. Following EGF treatment for 5 min, we observed strong phosphorylation of ERK and a significant nuclear localization of pERK. However, the nuclear localization of pERK gradually reduced with time. At 1 h of EGF stimulation, the nuclear localization of pERK is significantly reduced (Fig. 6). In the control experiments, following SD activation of EGFR in CHO-EGFR cells, the subcellular distribution of pERK followed a pattern in between that of PM and EN activation of EGFR, but the pattern is more similar to EN activation of EGFR. These data suggest that the location of EGFR activation determined the location where ERK is phosphorylated, which in turn influenced the subcellular trafficking of pERK. PM activation of EGFR resulted in the phosphorylation of ERK at and near the plasma membrane, which may slow the process of its translocation to the nucleus. On the other hand, EN activation of EGFR resulted in the phosphorylation of ERK near the perinuclear region, which may facilitate the nuclear translocation of pERK.

### Location-specific MEK activation controls spatio-temporal dynamics of ERK phosphorylation

**Figure 7:**
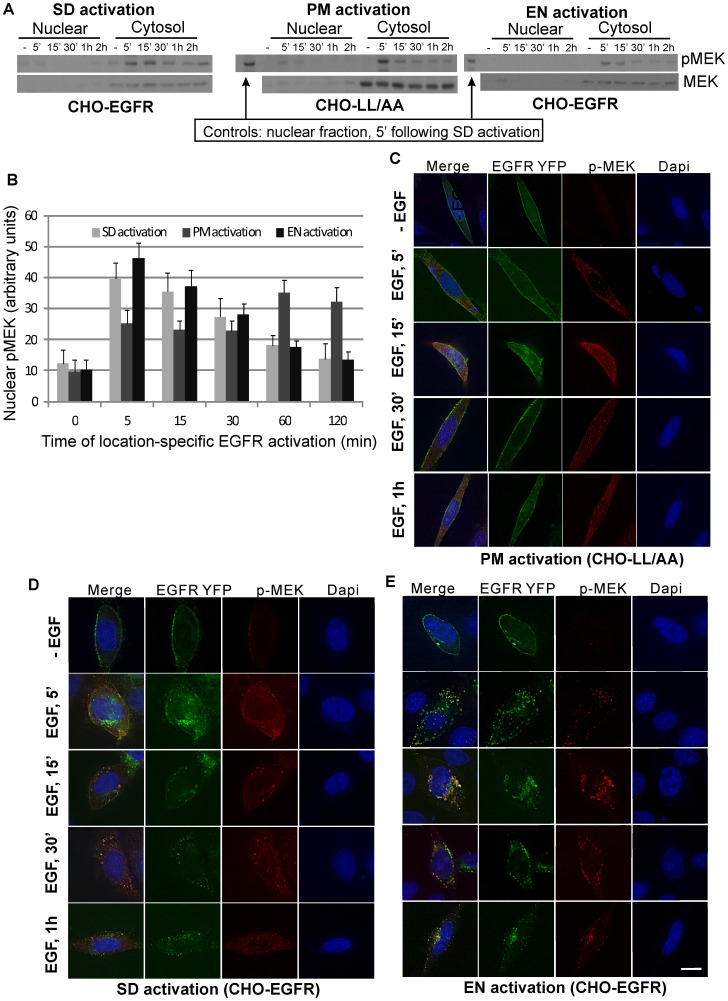
Spatio-temporal activation of MEK. (A) Following location-specific activation of EGFR for the indicated times, the spatio-temporal activation of MEK was determined by subcellular fractionation followed by immunoblotting with anti-pMEK antibody as described in Experimental procedures. (B) Quantification of MEK activation with the data from (A). MEK activation level is normalized against the level of total MEK. Each value is the average of at least three independent experiments and the error bar is the standard error. (C–E) Activation and translocation of MEK following location-specific EGFR activation. The activation and translocation of MEK (red) were examined by indirect immunofluorescence with antibody to pMEK followed by TRITC conjugated secondary antibody. EGFR localization (green) was revealed by the intrinsic fluorescence of tagged YFP and the nucleus was stained by Dapi (blue). Size bar, 10 µm.

We then focused on the molecular mechanisms by which the location-specific activation of EGFR controls the spatio-temporal dynamics of ERK activation. One important factor involved is the location where ERK is phosphorylated. It is likely that the location of ERK phosphorylation is controlled by the subcellular localization of its upstream activators such as MEK. We examined the subcellular location of pMEK, a direct ERK activator, in response to location-specific EGFR activation by both subcellular fractionation (Fig. 7A&B) and immunofluorescence (Fig. 7C, D, E). As shown in Fig. 7A&B, following both the PM and EN activation of EGF, MEK was strongly phosphorylated and the pMEK was enriched in the cytoplasmic fractions, not the nuclear fractions. However, immunofluorescence revealed that pMEK showed a different spatio-temporal distribution within the cytoplasm following PM activation from EN activation of EGFR (Fig. 7C, D, E). Following PM activation, pMEK was localized to PM and the adjacent regions for the first 30 min; at 60 min, pMEK localized to both the perinuclear and peripheral regions of the cell. In contrast, EN activation of EGFR led to the strong phosphorylation of MEK and the co-localization of pMEK and EGFR in the perinuclear region during the 1 h experimental period. In control, following SD activation of EGFR we observed initial plasma membrane localization of pMEK (5–15 min) and later perinuclear and endosomal localization of pMEK.

The spatio-temporal dynamic of pMEK further indicates that PM activation of EGFR leads the phosphorylation of ERK at or near the PM, and EN activation of EGFR results in the phosphorylation of ERK at or near EN in the perinuclear region.

### The spatio-temporal regulation of ELK1 and RSK activation by pERK

**Figure 8:**
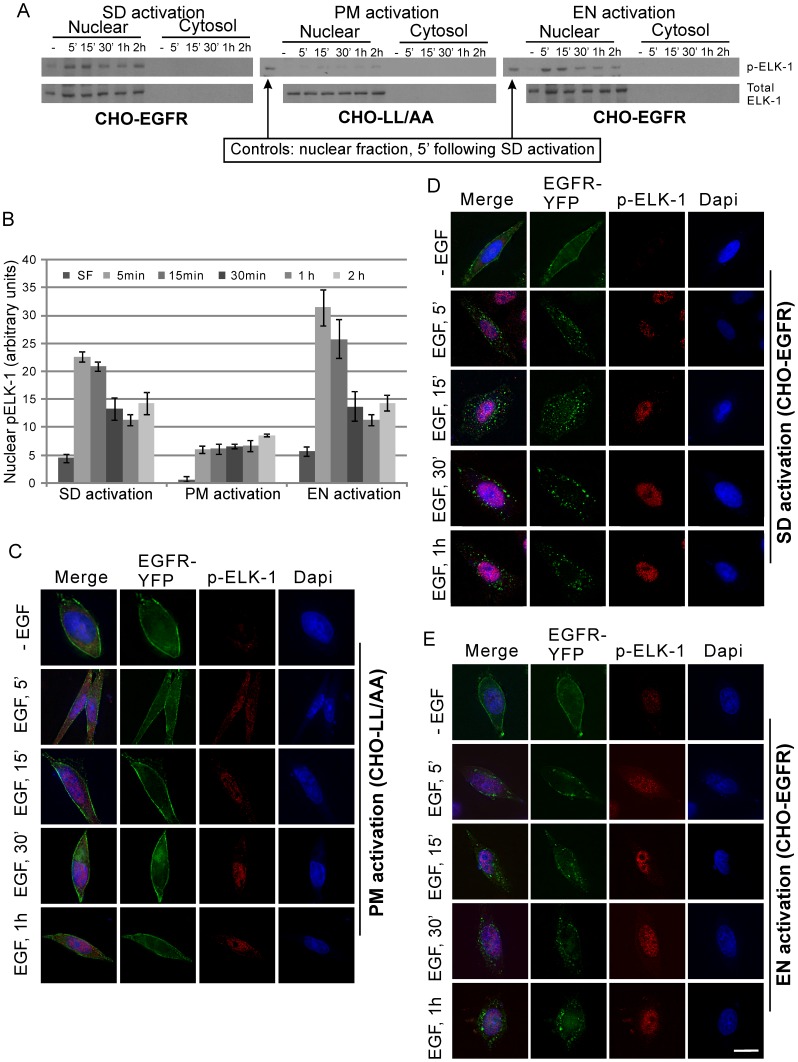
Spatio-temporal activation of ELK-1. (A) Following location-specific activation of EGFR for indicated time, the spatio-temporal activation of ELK-1 was determined by subcellular fractionation followed by immunoblotting with anti-pELK-1 antibody as described in Experimental procedures. (B) Quantification of ELK-1 activation with the data from (A). Each value is the average of at least three independent experiments and the error bar is the standard error. (C–E) Activation and translocation of ELK-1 following location-specific EGFR activation. The activation and translocation of ELK-1 (red) were examined by indirect immunofluorescence with antibody to pELK-1 followed by TRITC conjugated secondary antibody. EGFR localization (green) was revealed by the intrinsic fluorescence of tagged YFP and the nucleus was stained by Dapi (blue). Size bar, 10 µm.

To understand how the spatio-temporal distribution of pERK may affect the downstream signaling pathways and the activation and expression of c-jun and c-fos, we looked at the two direct substrate of pERK: ELK1 and RSK. Elk1is a direct substrate of pERK and has been shown to mostly localize in the nucleus [46], [47]. As shown in Fig. 8A, both SD and EN activation of EGFR in CHO-EGFR cells resulted in strong phosphorylation of ELK1 in nuclear faction at 5 min. The ELK1 phosphorylation then began to gradually decline. Importantly, no cytosolic ELK1 and pELK1 were detected both before and after EGF stimulation. On the other hand, PM activation of EGFR resulted in very weak ELK1 activation in the nucleus, and the level of activation slowly increased for a long period of up to 2 hours (Fig. 8A&B). This result was further examined by immunofluorescence. As shown in Fig. 8C, in CHO-LL/AA cells following PM activation of EGFR, ELK1 was weakly phosphorylated in the nucleus. On the other hand, in CHO-EGFR cells, p-ELK1 was exclusively localized in the nucleus following SD and EN activation of EGFR (Fig. 8D&E).

**Figure 9:**
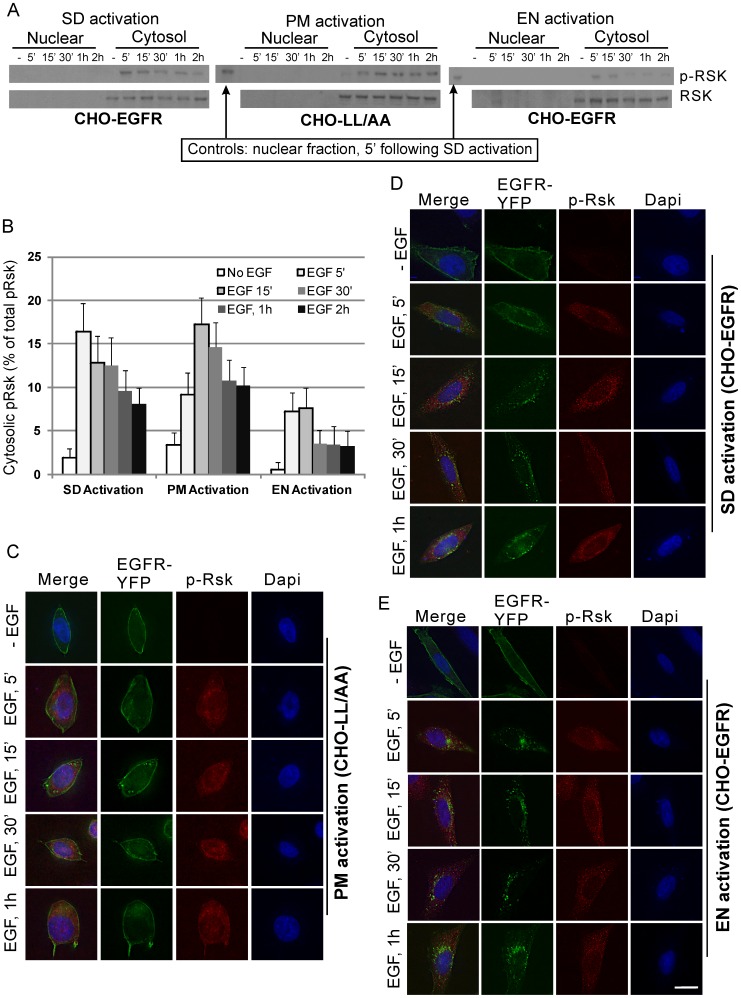
Spatio-temporal activation of RSK. (A) Following location-specific activation of EGFR for the indicated times, the spatio-temporal activation of RSK was determined by subcellular fractionation followed by immunoblotting with anti-pRSK antibody as described in Experimental procedures. (B) Quantification of RSK activation with the data from (A). Each value is the average of at least three independent experiments and the error bar is the standard error. (C–E) Activation and translocation of RSK following location-specific EGFR activation. The activation and translocation of RSK (red) were examined by indirect immunofluorescence with antibody to pRSK followed by TRITC conjugated secondary antibody. EGFR localization (green) was revealed by the intrinsic fluorescence of tagged YFP and the nucleus was stained by Dapi (blue). Size bar, 10 µm.

We next examined activation of RSK, a substrate of pERK mainly localized in cytosol [48], [49]. RSK activation was determined under all three treatment conditions in CHO-EGFR cells and CHO-LL/AA cells. As shown in Fig. 9A&B, both PM and EN activation of EGFR caused the phosphorylation of RSK at 5 min with a level of intensity that was comparable to the RSK activated following SD activation of EGFR. Immunofluorescence experiments were also conducted to examine the localization of pRSK. As shown in Fig. 9C, D, E, in all three experimental groups examined, RSK was similarly phosphorylated and the phosphorylated RSK mainly localized to the cytoplasm as expected.

### Effects of PMA on the spatio-temporal activation of ERK, ELK1 and RSK

**Figure 10:**
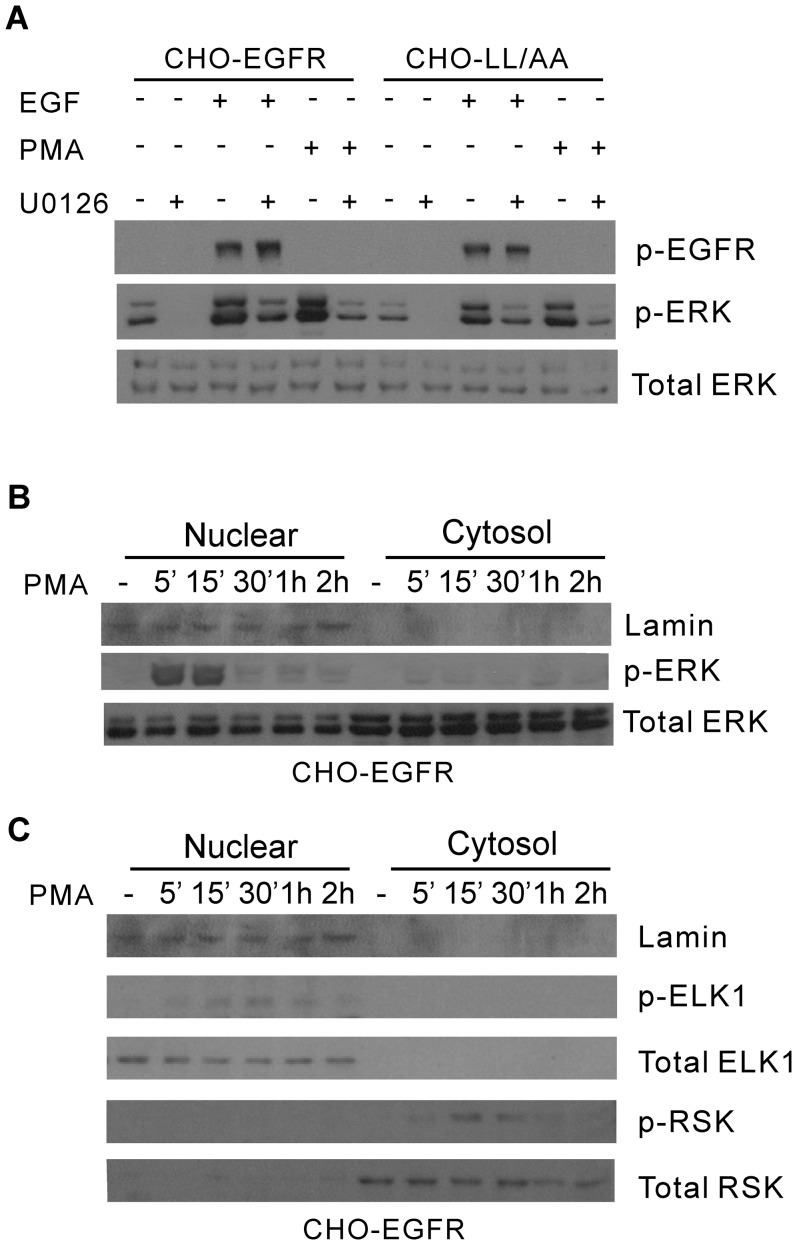
Activation of ERK and downstream signaling proteins ELK-1 and RSK by PMA. (A) CHO-EGFR and CHO-LL/AA cells were stimulated with PMA (10 nM) or EGF (100 ng/ml) with or without MEK inhibitor U1026 (10 µM) for 15 min. The activation of ERK was determined by immunoblotting with antibody to pERK. (B) Nuclear localization of pERK following PMA stimulation. The CHO-EGFR cells were stimulated with PMA (10 nM) for the indicated times. The cell homogenates were subcellularly fractionated into nuclear and non-nuclear fractions as described in Experimental procedures. The level of pERK was determined by immunoblotting with anti-pERK antibody. (C) The activation and nuclear localization of ELK1 and RSK. The CHO-EGFR cells were stimulated with PMA (10 nM) for the indicated times. The cell homogenates were subcellular fractionated into nuclear and non-nuclear fractions. The level of ELK-1 and RSK activation was determined by immunoblotting with anti-pELK-1 and pRSK antibodies.

To further examine whether the spatio-temporal activation of ERK contributes to the specific activation of downstream signaling proteins and transcription factor, we treated the cells with PMA to generate specific ERK activation in the nucleus. It has been shown that PMA treatment resulted in the accumulation of pERK in the nucleus [50]. Indeed we showed that treatment with PMA resulted in very strong ERK phosphorylation similar to EGF stimulation in both CHO-EGFR and CHO-LL/AA cells (Fig. 10A). Furthermore, for CHO-EGFR cells the pERK induced by PMA was mostly localized in the nucleus in a pattern very similar to EN activation of EGFR (Fig. 10B). We then examined the effects of nuclear pERK on the activation of ELK1 and RSK by subcellular fractionation and immunoblotting. As shown in Fig. 10C, the pattern of ELK1 and RSK activation following PMA treatment is very similar to that following EN activation of EGFR. PMA treatment resulted in ELK1 phosphorylation in the nucleus. No cytosolic ELK1 and pELK1 were detected both before and after PMA stimulation. On the other hand, PMA treatment caused the phosphorylation of RSK that was mostly localized in the cytoplasm. Together, these data suggest that spatio-temporal dynamics of pERK play an important role in the activation of downstream signaling molecules ELK1 and RSK.

### Effects on cell size and cell proliferation

**Figure 11:**
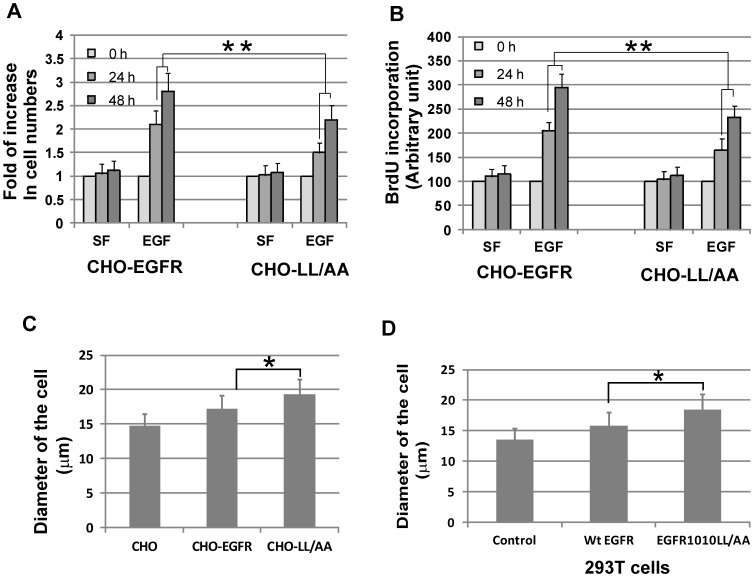
The effects on cell proliferation and cell size. (A–B) the effects of location-specific activation of EGFR on cell proliferation. CHO-EGFR and CHO-LL/AA cells were stimulated with EGF (50 ng/ml) for the indicated times. the cell proliferation was measured by the fold of increase of the cell numbers (A) or by the BrdU incorporation (B). (C–D) The effects of location-specific EGFR activation on cell size. For C, the cell sizes of CHO-EGFR and CHO-LL/AA cells were measured and compared. For D, the 293T cells were transiently transfected with either the YFP-tagged wild type EGFR or mutant EGFR1010LL/AA. Three days later, the cell sizes were measured and compared. The cell sizes were determined by the diameter of the cells. The calculation of the diameter was described in Materials and Methods. * indicates that the difference is significant (P<0.05). ** indicates that the difference is very significant (P<0.01).

We finally examined the effects of location-specific EGFR signaling on the physiological outcomes of the cells. We first examined the effects on cell proliferation. We compared EGF induced cell proliferation in CHO-EGFR and CHO-LL cells. Two methods were used to determine the cell proliferation rate, BrdU incorporation and cell counting. We showed by both methods that SD activation of EGFR by EGF in CHO-EGFR cells result in stronger cell proliferation than PM activation of EGFR by EGF in CHO-LL/AA cells and the differences are statistically significant (Fig. 11A&B).

We also examined the effects of long-term EGFR signaling from PM on cell size. We compared the cell size of CHO, CHO-EGFR and CHO-LL/AA cells by measuring the cell diameter. As shown in Fig. 11C, CHO-LL/AA cells have larger cell size than CHO-EGFR cells. The cell size of the parent CHO cells is smaller than both of the two selected stable cell lines. To further examine whether the difference in cell size is due to the expression of wild type and endocytosis-deficient EGFR or the selection of the cell clones, we transiently transfected 293T cells with wild type EGFR or EGFR-1010LL/AA. The cell sizes were measured three days later following the transfection. As shown in Fig. 11D, expression of EGFR1010LL/AA results in larger cell size than the expression of wild type EGFR. Together, these data suggest that PM EGFR signaling may cause a larger cell size.

## Discussion

It is well established that endocytosis and trafficking of membrane receptors are vital in providing spatio-temporal control of receptor-mediated cell signaling. However, little is known about the dynamics of spatio-temporal activation of vital signaling molecules such as EGFR and ERK. It is not clear whether and how the activation of membrane receptors at different subcellular locations and at different times affect downstream cell signaling and physiological outcomes. The lack of progress is partially due to the lack of a proper system that allows the location-specific activation of membrane receptors. We previously established a system that allows the specific activation of EGFR in endosomes [22], [23], [39]. We also identified an endocytosis-deficient mutant EGFR1010LL/AA with mutation of the dileucine motif ^1010^LL^1011^ to alanines [42]. Since the mutation lies outside of the kinase domain and not at the tyrosine phosphorylation sites, this mutant may be fully activated by EGF and generate persistent EGFR signaling only at PM. Here, we demonstrated that we have established a system to allow the specific activation of EGFR either at PM or in EN using our previously generated two stable cell lines CHO-EGFR and CHO-LL/AA. We showed that we can specifically activate EGFR at the PM by stimulating CHO-LL/AA cells with EGF, and we can specifically activate EGFR in the EN using our previously established protocol [22] (Fig. 1). The activation is very location-specific as revealed by both immunofluorescence and subcellular fractionation analysis (Fig. 1). The successful establishment of such a system to specifically activate EGFR at PM and EN is very significant. Firstly, the system to specifically activate EGFR in EN as we reported previously [22] and reported here for CHO-EGFR cell is the only system to activate EGFR in EN without EGFR activation at PM. Secondly, the system we reported here to specifically activate EGFR at PM is better than previously reported systems. Previously, the efforts to create the specific PM activation of EGFR were focused on targeting the endocytic machinery proteins such as dynamin and clathrin, which resulted in the broad inhibition of endocytosis. In our system only EGFR endocytosis is specifically blocked. Thirdly, the establishment of a system to specifically activate EGFR at PM and in EN allows, for the first time, a direct comparison of EGFR signaling between PM and EN.

In order to achieve specific activation of EGFR at the plasma membrane by using the endocytosis-deficient EGFR1010LL/AA, we need to express this mutant in cells that does not express endogenous EGFR. As most epithelial cells express more or less EGFR, we chose to use CHO cells. CHO cells have been used previously to study EGFR signaling after being transfected with various mutant EGFR expression vectors [51], [52]. These previous study showed that various EGFR signaling pathways including ERK pathway is well activated following the stimulation of EGFR by EGF in CHO cells [51], [52].

Using this system, we showed that the activation of EGFR at the PM and EN results in a similar phosphorylation of EGFR in terms of phosphorylation sites and intensity (Fig. 2). We further showed that location-specific activation of EGFR results in the similar association of EGFR with many downstream binding proteins including Grb2, SHC, PLC-γ1, p120 rasGAP, p85 and Cbl (Fig. 3A). The only noticeable difference is that EN activation of EGFR induced a weaker phosphorylation at Y1086 than PM activation of EGFR (Fig. 2), and PLC-γ1 bound to activated EGFR following PM activation of EGFR more strongly than that following EN activation of EGFR (Fig. 3A). We do not know why PM activation of EGFR results in stronger activation of Y1086. However, the stronger Y1086 phosphorylation could explain the stronger binding of EGFR with PLC-γ1 following PM activation of EGFR as Y1086 is one of the phosphoretyrosine sites that bind to PLC-γ1 [53].

In this research, the cells were treated with EGF at 100 ng/ml. While this dosage is much higher than the physiological level of EGF, this is the dosage used in most research labs to study EGFR-mediated cell signaling and endocytosis. It has also been reported that EGFR endocytosis may follow different pathway under different EGF concentration [54]. It has been shown that EGFR endocytosis is through clathrin-mediated endocytosis at low dosage of EGF (1.5 ng/ml) and through both clathrin-mediated and non-clathrin-mediated endocytosis at higher dosages [54]. However, we recently showed that EGFR endocytosis followed the same pathway at various EGF concentration ranging from 2 to 100 ng/ml [55]. Therefore, it is likely that our findings may also apply to lower EGF dosage. An advantage of using high EGF concentration in our system is that it causes rapid and nearly complete EGFR internalization into EN to allow strong EN activation of EGFR.

By comparing the effects of PM and EN activation of EGFR, we had some interesting findings. The level and pattern of total phosphorylated Erk1/2 are very similar following PM and EN activation of EGFR (Fig. 3B&C). However, the phosphorylation and expression of transcription factors c-jun and c-fos are differentially regulated by PM and EN activation of EGFR (Fig. 4). The results regarding the activation of ERK by location-specific EGFR activation are very controversial [29]–[32]. Some data show that endosomal EGFR signaling is essential in ERK activation as inhibiting EGFR endocytosis resulted in the inhibition of ERK activation [29]. But, other data show that the inhibition of EGFR endocytosis actually enhances ERK activation [32], [56]. Our data demonstrate that either PM or EN activation of EGFR is sufficient to activate ERK, which suggests that EGFR endocytosis is not required for the activation of ERK by EGF. However, location-specific activation of EGFR does regulate ERK-mediated cell signaling through controlling the spatio-temporal dynamics of pERK.

We showed that PM activation of EGFR results in slow and lasting buildup of pERK in nucleus, but EN-activation of EGFR results in strong but transient build up of pERK in nucleus (Fig. 5 & 6). Our data suggest that the spatio-temporal dynamics of pERK is at least partially determined by the spatio-temporal dynamics of its upstream activators pMEK (Fig. 7). Following PM activation, pMEK was localized to the plasma membrane and adjacent regions for the first 30 min, then at 60 min, pMEK localized to both perinuclear and peripheral region of the cell. In contrast, EN activation of EGFR led to the strong phosphorylation of MEK and the co-localization of pMEK and EGFR in the perinuclear region. These data suggest that EN activation of EGFR results in the activation of pMEK in the perinuclear region where pMEK activates ERK, with activated ERK then quickly translocating to the nucleus. As the activated EGFR in endosomes gradually trafficked to lysosome for degradation, the level of pMEK and pERK decreased gradually. On the other hand, PM activation of EGFR results in the activation of pMEK near the PM where pMEK activate ERK. The pMEK continue to activate ERK while diffusing to the perinuclear region. The pERK gradually move to the nucleus by various mechanisms including diffusion, as previously described. As activated EGFR is not internalized and keep signalling from PM, we observed the sustained build up of pERK in nucleus.

It is interesting to find out how the location-specific activation of EGFR regulates spatio-temporal dynamic of pMEK. One possibility is that the location-specific activation of MEK is controlled by its upstream activator Raf1. As Raf1 must be targeted to the membrane to be activated [57], it is possible that phospho-Raf1 (pRaf1) is associated with the PM mostly following PM activation and associated with endosome membrane mostly following EN activation of EGFR. Further research is needed to determine this.

The spatio-temporal dynamics of pERK could also be regulated by many other mechanisms following location-specific activation of EGFR. For example, phosphatases may be involved in controlling the spatio-temporal dynamics of the ERK cascade. This issue is recently listed as one of the important pending issues that needs to be resolved in ERK dynamics [58]. There are ten MAPK phosphatases (MKPs) that act as negative regulators of MAPK activity in mammalian cells [59].

Our data also suggest a possible mechanism for location-specific activation of EGFR to differentially regulate transcription factors c-jun and c-fos. We showed that EN activation of EGFR results in the strong activation of ELK1 in the nucleus. It is well established that ELK1 is an ERK substrate and is mostly localized in the nucleus to function as a transcription factor. As EN activation of EGFR results in strong and quick accumulation of pERK in nucleus (Fig. 8), it is logical to see the nuclear-localized ELK1 is phosphorylated strongly. On the other hand, PM activation of EGFR result in weak and persistent phosphorylation of ELK1 (Fig. 8), possibly due to the relatively weak and slow buildup of pERK in nucleus (Fig. 5&6). We also showed that PM activation of EGFR results in stronger RSK phosphorylation in cytoplasm than EN activation of EGFR (Fig. 9). As RSK is an upstream activator of c-fos [60], it is likely that stronger c-fos activation induced by PM activation of EGFR is through the activation of RSK. The stronger RSK phosphorylation could be due to the longer residential time of pERK in the cytosol following PM activation of EGFR (Fig. 5&6).

To confirm that the spatio-temporal dynamics of pERK is responsible for the differential activation of c-jun and c-fos, we attempted to use a different way to generate specific spatio-temporal pattern of pERK. It has been shown that PMA treatment resulted in the accumulation of pERK specifically in the nucleus [53]. Thus, we treated the cells with PMA to specifically accumulate pERK in the nucleus and examined whether this nuclear accumulation of pERK specifically activates ELK1 in the nucleus. Indeed, PMA stimulation results in the accumulation of pERK in the nucleus, the strong activation of nuclear ELK1, but not cytosolic RSK (Fig. 10).

It is difficult but important to examine the physiological outcomes of location-specific activation of EGFR. We showed here that PM activation of EGFR is less potent than SD activation of EGFR in terms of EGF-stimulated cell proliferation (Fig. 11A&B). We showed previously that EN activation of EGFR stimulates cell proliferation to a similar level as SD activation of EGFR [23]. These data suggest that EGFR signaling from PM may have less influence on cell proliferation than EGFR signaling from EN. We also examined the effects of location-specific EGFR activation on cell size. Interestingly, PM activation of EGFR increased cell size (Fig. 11C&D). Further research is needed to understand the mechanisms behind this.

It is also interesting to notice that in general the effects of EN signaling of EGFR more resembles SD signaling of EGFR than PM signaling of EGFR. One explanation is that during SD activation of EGFR, activated EGFR only localizes at the PM for a few minutes, but localizes in endosomes for one to two hours. This finding highlights the importance of EN EGFR signaling.

We may gain more insight by comparing the data about EGFR with the data about other receptor tyrosine kinases (RTKs). The role of trafficking on cell signaling and function has also been studied in many other RTKs including nerve growth factor receptor (Trks), vascular endothelial growth factor receptor (VEGFR), and hepatocyte growth factor receptor (c-Met) [61]–[68]. Perturbation of endocytosis by the depletion of either clathrin heavy chain or an ESCRT-0 subunit caused differential effects on ligand-stimulated VEGFR2 proteolysis and signaling [61]. Internalization does not terminate its signaling; instead, the internalized VEGFR2 is phosphorylated, co-distributes with active phospholipase C-gamma, and stimulates p44/42 mitogen-activated protein kinase phosphorylation and cell proliferation [64]. Interestingly, it was shown that cell proliferation is limited by retaining VEGFR-2 at the membrane [64], which is consistent with our finding about EGFR. It has also been reported that c-Met signaling is closely regulated by its location [62], [63]. To activate STAT3, the activated c-Met itself needs to be delivered to a perinuclear endosomal compartment to sustain phosphorylated STAT3 in the nucleus. This is signal specific because c-Met–induced extracellular signal-regulated kinase nuclear accumulation does not require receptor trafficking to the perinuclear compartment. This response is triggered from peripheral endosomes. Thus, control of growth factor receptor traffic determines the nature of the signal output [63]. Nerve system provides an excellent model in studying the effects of location-specific RTK signaling, generated through receptor endocytosis, on cell functions as the PM (nerve ending) could be physically far away from endosomes (in cell body). In neurons, many receptors must be localized correctly to axons or dendrites for proper function. During development, receptors for nerve growth and guidance are targeted to axons and localized to growth cones where receptor activation by ligands results in promotion or inhibition of axon growth. Signaling outcomes downstream of ligand binding are determined by the location, levels and residence times of receptors on the neuronal plasma membrane. Therefore, the mechanisms controlling the trafficking of these receptors are crucial to the proper wiring of circuits [69]. Indeed, it has been reported that Trk retrograde signaling requires the internalization of Trk into Pinch-directed endosomes, and Overexpression of Pincher increases the internalization of TrkA, and enhances recruitment of phospho-ERK5 but not Erk1/2, after addition of NGF [67], [70].

In conclusion, we have established a system to allow the specific activation of EGFR at different subcellular locations: PM and EN. Our data indicate that the location-specific EGFR activation differentially regulate various transcription factors including c-jun, c-fos and ELK1 by controlling the spatio-temporal activation of ERK. The EGFR signaling from different cellular locations also affect the physiological outcome of the cells.
