# Correction: Differential Regulation of Transcription Factors by Location-Specific EGF Receptor Signaling via a Spatio-Temporal Interplay of ERK Activation

**DOI:** 10.1371/annotation/2a7e4838-1938-4728-b796-f4cf06b8c36d

**Published:** 2013-01-29

**Authors:** Peng Wu, Ping Wee, Jennifer Jiang, Xinmei Chen, Zhixiang Wang

Due to an error, the graphs in Figure 5B and 7B were duplicated. Thus, we are making a corrected Figure 7B available here: 

**Figure:**
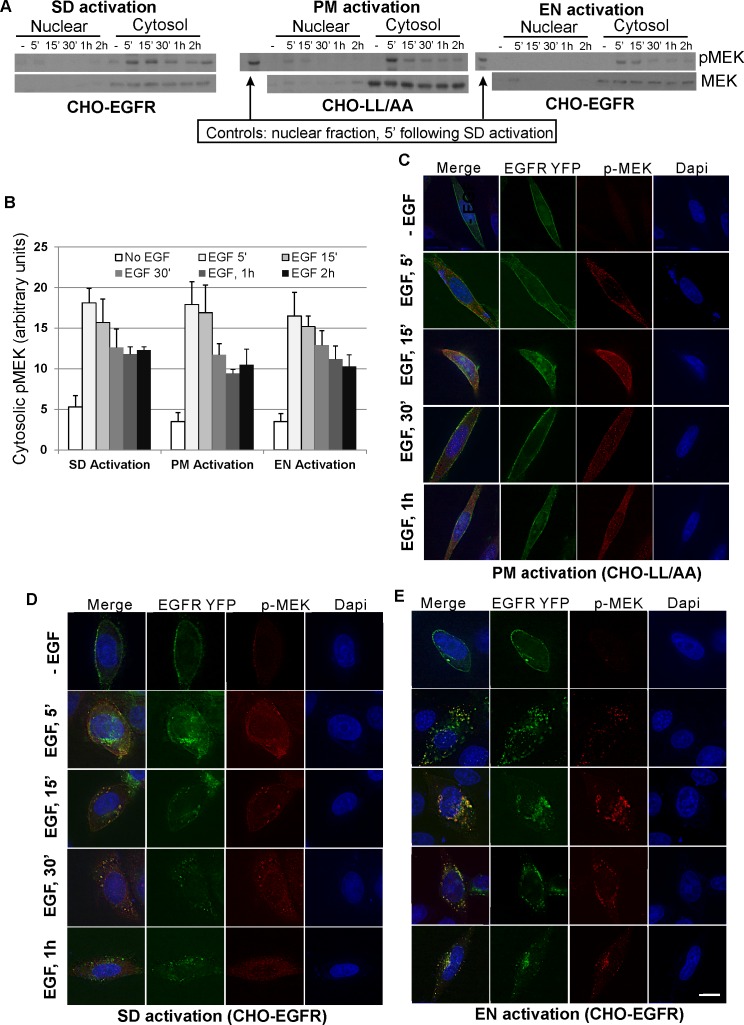


However, the description of the results, as reported in the article, is not affected by this error.

